# „Akutstationäre Rheumatologie“ in Deutschland: Unverzichtbare Säule in der rheumatologischen Versorgung und der Facharztweiterbildung

**DOI:** 10.1007/s00393-025-01714-z

**Published:** 2025-09-02

**Authors:** Alexander Pfeil, Martin Fleck, Hanns-Martin Lorenz, Ulf Müller-Ladner, Heinz-Jürgen Lakomek, Christof Specker

**Affiliations:** 1 Rheumazentrum (G-BA-Kriterien) sowie Sektion Rheumatologie und Osteologie, Klinik für Innere Medizin III, Universitätsklinikum Jena, Am Klinikum 1, 07747 Jena, Deutschland; 2 Klinik und Poliklinik für Innere Medizin I, Universitätsklinikum Regensburg, Regenburg, Deutschland; 3 Klinik und Poliklinik für Rheumatologie/Klinische Immunologie, Asklepios Klinikum Bad Abbach, Bad Abbach, Deutschland; 4 Sektion Rheumatologie, Medizinische Klinik V, Universitätsklinikum Heidelberg, Heidelberg, Deutschland; 5 Abteilung für Rheumatologie & Klinische Immunologie, Campus Kerckhoff, Justus-Liebig-Universität Gießen, Gießen, Deutschland; 6 Johannes Wesling Klinikum Minden, Minden, Deutschland; 7 Klinik für Rheumatologie und Klinische Immunologie, Evangelisches Krankenhaus Kliniken Essen-Mitte, Essen, Deutschland

**Keywords:** Rheumatologie, Stationäre Versorgung, Weiterbildung, Rheumatology, Inpatient care, Advanced training

## Abstract

Die Auswirkungen der aktuellen Krankenhausreform wie auch die zunehmende Ambulantisierung der medizinischen Versorgung in Deutschland werden zu einem Rückgang stationärer Weiterbildungskapazitäten führen, womit aktuell wie auch zukünftig fachärztliche Versorgungsengpässe auch in der ambulanten Patientenversorgung drohen. Dieses Szenario könnte auch die Rheumatologie treffen, obwohl die Prävalenz als auch die Komplexität entzündlich rheumatischer Erkrankungen mit systemischer Organbeteiligung eine flächendeckende akutstationäre rheumatologische Versorgung dringend erforderlich machen. Die medizinische, strukturelle und weiterbildungsbezogene Notwendigkeit einer stationären rheumatologischen Versorgung ergibt sich nicht nur durch die Einführung innovativer Therapiekonzepte, sondern insbesondere auch aufgrund hoch-entzündlicher und schwer verlaufender entzündlich rheumatischer Erkrankungen mit Multiorganbeteiligung wie auch aufgrund vielfältiger Komorbiditäten oder der Behandlung von älteren Menschen mit Polypharmaziemultimorbidität. Die geplante Überarbeitung der Musterweiterbildungsordnung muss daher auch im Fachgebiet der Rheumatologie weiterhin einen sachgerechten Anteil stationärer Weiterbildung berücksichtigen, eine gezielte Finanzierung von fachärztlicher Weiterbildung im Rahmen der Vorhaltepauschalen des Krankenhausversorgungsverbesserungsgesetzes (KHVVG) ist vorgesehen.

## Abstract (en)

The effects of the current hospital reform as well as the increasing shift from inpatient care to outpatient care in Germany will lead to a decline in inpatient training capacities, with the threat of current and future shortages of specialists in outpatient care. This scenario could also affect rheumatology, although the prevalence and complexity of inflammatory rheumatic diseases with systemic organ involvement make comprehensive acute inpatient rheumatology care urgently necessary. The medical, structural and training-related need for inpatient rheumatological care arises not only from the introduction of innovative treatment concepts but also in particular due to highly inflammatory and severe systemic rheumatic diseases with multiorgan involvement as well as from a wide range of comorbidities or the treatment of older people with polypharmacy multimorbidity. The planned amendment of the model advanced training regulations for rheumatism should therefore continue to take an appropriate proportion of inpatient training into account and targeted funding of specialist education is planned as part of the flat-rate payments under the Hospital Care Improvement Act (KHVVG).

Die vormalige und aktuelle Krankenhausreform in Deutschland sowie die zunehmende Ambulantisierung der medizinischen Versorgung haben zu einem Rückgang stationärer Weiterbildungskapazitäten in vielen Fachbereichen der Inneren Medizin wie auch der Rheumatologie geführt. Auf Basis des Memorandums 2024 der Deutschen Gesellschaft für Rheumatologie und Klinische Immunologie (DGRh) wird ein Mehrbedarf von 700 Fachärztinnen und Fachärzten für Innere Medizin und Rheumatologie gesehen [1]. Parallel hierzu steigen sowohl die Prävalenz als auch die Komplexität entzündlich rheumatischer Erkrankungen. Dieser Übersichtsartikel beleuchtet die medizinische, strukturelle und ausbildungsbezogene Notwendigkeit stationärer rheumatologischer Versorgung und Weiterbildung. Die Versorgung schwerer Verläufe und komplexer immunologischer Systemerkrankungen wie auch die Einführung innovativer Therapiekonzepte und die Behandlung bei komplexer Multimorbidität erfordern eine gezielte Sicherung stationärer Strukturen im Fachgebiet Rheumatologie, auch und gerade im Hinblick auf die geplante Überarbeitung der Musterweiterbildungsordnung.

## Der Wandel rheumatologischer Krankheitsbilder: Von Gelenkerkrankung zur Systemerkrankung

Entzündlich rheumatische Erkrankungen, traditionell als Erkrankungen des Bewegungsapparates wahrgenommen, sind in ihrer heutigen klinischen Realität vielmehr komplexe immunologische Systemerkrankungen mit potenzieller Multiorganbeteiligung [1]. Sie zeichnen sich durch eine akute oder chronisch rezidivierende, systemische Inflammation aus, die neben den Gelenken auch innere Organe wie Lunge, Herz oder Niere betreffen kann [2].

Die demografische Entwicklung mit zunehmendem Anteil älterer Menschen in der Bevölkerung führt zu einem Anstieg der Prävalenz rheumatischer Erkrankungen: Von ca. 1,45 Mio. Betroffenen im Jahr 2016 [3] wird ein Anstieg auf 1,5 bis über 2 Mio. Erkrankte in Deutschland erwartet, was einer Prävalenz von 2,2–3 % der erwachsenen Bevölkerung entspricht [4]. Damit steigen nicht nur die Fallzahlen, sondern auch die klinische Komplexität sowie die Anforderungen an die Versorgungsstrukturen.

Entzündlich rheumatische Erkrankungen haben zudem eine hohe volkswirtschaftliche Relevanz – v. a., wenn aufgrund unzureichender Versorgung irreversible Schäden entstehen. Diese können beispielsweise zu einer eingeschränkten Gelenkfunktion oder zu Organschäden führen. In der Folge kann die Arbeitskraft der Patienten beeinträchtigt werden, und es entstehen möglicherweise weitere Gesundheitskosten. Solche Schädigungen können auch durch eine verspätete Diagnose verursacht werden. Dies ist ein weiterer Grund für eine flächendeckend kompetente rheumatologische Versorgung und eine zügige diagnostische Abklärung, die zum Teil nur im stationären Setting optimal durchgeführt werden kann.

## Innovative Systemtherapien erfordern stationäre Rahmenbedingungen

In Abhängigkeit von der Ausprägung entzündlich rheumatischer Erkrankungen ist eine Beteiligung aller Organsysteme (z. B. Lunge, Herz und Niere) möglich [2]. Die akute Beteiligung eines morbiditäts- und mortalitätsrelevanten Organs, wie beispielsweise Lunge, Herz oder Nieren, im Rahmen einer entzündlich rheumatischen Erkrankung macht oft eine stationäre Versorgung erforderlich, um zeitnah eine mitunter aufwendige Differenzialdiagnostik (z. B. Infektion unter Immunsuppression vs. Schub der Grunderkrankung) und eine oft intensive und komplexe immunsuppressive Therapie durchführen zu können. Unter Berücksichtigung der Multiorganbeteiligung bei entzündlich rheumatischen Erkrankungen sind intensivierte immunsuppressive Therapien heutzutage häufig, zudem finden neue innovative Therapieformen den Weg in die Klinik wie chimäre Antigenrezeptor(CAR)-T-Zellen [5] oder bi- oder trispezifische T‑Cell-Engager (TCE) [6]. Diese Therapien sind mit erheblichen Nebenwirkungsrisiken verbunden, wie etwa Zytokinfreisetzungssyndromen oder Infektionskomplikationen [5, 6] und erfordern eine gute Schulung des pflegerischen und ärztlichen Personals mit engmaschiger Überwachung und interdisziplinärer Steuerung – Anforderungen, die nur in stationären Einrichtungen erfüllt werden können. Der Wandel zu mehr entzündlichen Systemerkrankungen, differenzialdiagnostische Herausforderungen durch komplexere und akutere immunologische Mechanismen (wie z. B. der Autoinflammation) und die Einführung neuer Immuntherapien unterstreicht die essenzielle Rolle der stationären Versorgung im Kontext einer modernen Rheumatologie.

## Indikationen und Kontextfaktoren für stationäre Aufnahmen

Die Entscheidung zur stationären Aufnahme rheumatologischer Patientinnen und Patienten wird nicht allein durch die Grunderkrankung bestimmt, sie erfolgt unter Einbeziehung spezifischer Indikationen und Kontextfaktoren, die eine ambulante Versorgung entweder unmöglich oder medizinisch nicht vertretbar machen. Die zunehmende Komplexität entzündlich rheumatischer Systemerkrankungen und der Wandel hin zu personalisierten Therapiekonzepten macht ein differenziertes Indikationssystem für die stationäre Versorgung erforderlich.

### Indikationen für die stationäre Aufnahme

Im stationären Setting werden Patienten mit entzündlich rheumatischen Gelenkerkrankungen wie rheumatoider Arthritis, Spondyloarthritis und Psoriasisarthritis, mit Kollagenosen wie systemischem Lupus erythematodes sowie mit Vaskulitiden wie Riesenzellarteriitis und Granulomatose mit Polyangiitis behandelt [7]. Eine weitere häufige Indikation ist Fieber unklarer Genese oder unter Immunsuppression. Aufgrund der oft komplexen Diagnostik und folgenden Therapie sind häufig länger stationäre Aufenthalte notwendig, sodass im Rahmen des KOBRA(*K*ontinuierliches *O*utcome *B*enchmarking in der *R*heumatologischen *A*kutversorgung)-Projekts des Verbandes Rheumatologischer Akutkliniken (VRA) gezeigt wurde, dass Patienten mit komplexen entzündlich-rheumatischen Erkrankungen, wie z. B. systemischen Vaskulitiden, im Mittel 8,2 ± 7,4 Tage stationär behandelt werden [8].

Der VRA veröffentlichte 2021 eine indikationsbasierte Liste, welche die Notwendigkeit stationärer rheumatologischer Versorgung definiert. Zu den zentralen Indikationen gehören (*Details s. *Tab. 1; [9]):

- *akuter Schub einer entzündlich rheumatischen Erkrankung* mit drohender oder manifester Organbeteiligung (z. B. Lunge, Niere, Herz).
- *ambulant nicht ausreichend abklärbare oder beherrschbare Symptomatik*, insbesondere bei unklaren systemischen Symptomen mit Verdacht auf immunvermittelte Erkrankungen,
- *Einleitung oder Umstellung einer intensivierten immunsuppressiven Therapie*, etwa bei hoher Krankheitsaktivität oder Therapieversagen,
- *Therapiekomplikationen*, z. B. Infektionen unter Immunsuppression oder organspezifische Nebenwirkungen (Hepato- oder Nephrotoxizität, Zytopenien),
- *Behandlung multimorbider und geriatrischer Patienten* mit entzündlich rheumatischen Erkrankungen, bei denen eine ambulante Therapie nicht ausreichend koordiniert und sicher durchgeführt werden kann,
- *Multidisziplinäre Versorgung* in Fällen mit Systemerkrankungen oder Organmanifestationen, die die Einbindung weiterer Fachrichtungen (z. B. Nephrologie, Pulmologie, Infektiologie, Neurologie) erforderlich machen.

**Tab. 1:** Indikationsliste des Verbandes Rheumatologischer Akutklinik (VRA) zur stationären Aufnahme in die Rheumatologie. (Adaptiert nach Lakomek 2021 [9])

1.	Patienten mit komplexer Krankheitssymptomatik und Verdacht auf eine rheumatische Erkrankung bei bis lang ambulant nicht gelungener Diagnosestellung
2.	Entzündlich rheumatische Erkrankungen im ambulant nicht beherrschbaren akuten Schub
3.	Entzündlich rheumatische Erkrankungen mit drohender oder potenziell fortschreitender Organbeteiligung
4.	Ambulant nicht beherrschbare Schmerzzustände muskuloskeletaler Erkrankungen zur multimodalen konservativen Behandlung
5.	Behandlung gravierender Therapiekomplikationen, z. B. Organtoxizität
6.	Reevaluation bei komplexem Krankheitsverlauf mit Verschlechterungstendenz zur möglichen Neufestlegung einer differenzierten immunsuppressiven Therapie
7.	Stationäre Versorgung von Patienten mit rheumatischen Erkrankungen zur akuten Verbesserung funktioneller Defizite
8.	Spezielle systemische und lokale Gelenk- und Wirbelsäulentherapien, z. B. überwachungspflichtige Infusionen und Injektionen, auch bei relevanter funktioneller Beeinträchtigung
9.	Patienten mit rheumatischen Erkrankungen und klinisch relevanten Komorbiditäten, z. B. kardiovaskulären Erkrankungen oder Diabetes Typ 2
10.	Akutstationäre Versorgung multimorbider geriatrisch rheumatologischer Patienten zur Funktionsverbesserung und Optimierung der Therapieeinstellung bzw. Behandlung rheumatischer Erkrankungen im höheren Lebensalter
11.	Behandlung rheumatologischer Notfälle mit akuter oder drohender Organdysfunktion
12.	Behandlung von autoinflammatorischen Erkrankungen
13.	Behandlung von primären und sekundären Immundefekten
14.	Behandlung von schweren Infektionen unter immunsuppressiver Therapie oder bei Immundefekten

Außerdem sollten, unter Berücksichtigung der Weiterentwicklungen im Fachgebiet der Rheumatologie und klinischen Immunologie, die vom VRA 2021 formulierten Punkte zur stationären Aufnahme um die Behandlung von rheumatologischen Notfällen mit akuter oder drohender Organdysfunktion, von akuten autoinflammatorischen Erkrankungen sowie von primären und sekundären Immundefekten ergänzt werden. Hierzu zählen ebenfalls die Behandlung von schweren Infektionen unter immunsuppressiver Therapie sowie bei Immundefekten (s. Tab. 1). Neu müssen die innovativen, eine stationäre Einleitung erfordernden Behandlungsformen (CAR-T-Zellen, bi-/trispezifische TCE) gelistet werden.

### Kontextfaktoren als Erweiterung des Indikationskatalogs

Neben den klassischen medizinischen Indikationen spielen Kontextfaktoren eine zunehmend wichtige Rolle bei der Entscheidung zur stationären Versorgung. Diese Faktoren umfassen sowohl klinische als auch soziale und funktionelle Aspekte, die eine umfassende stationäre Betreuung begründen. Zu den zentralen Kontextfaktoren zählen (Details s. Tab. 2):

- *hohe Krankheitsaktivität*, die ambulant nicht ausreichend kontrollierbar ist,
- *starke Schmerzsymptomatik*, welche eine zeitnahe multimodale Schmerztherapie erfordert,
- *funktionelle Einschränkungen* wie akute Verschlechterung der Selbsthilfefähigkeit, Mobilität, kognitiven oder emotionalen Fähigkeiten – insbesondere bei älteren Patientinnen und Patienten,
- *Erstmanifestation oder Rezidiv einer systemischen Erkrankung*, z. B. Vaskulitis oder Kollagenose, mit unklarer Organbeteiligung,
- *Vorliegen von Multimorbidität*, bei der eine engmaschige Überwachung oder Therapiekoordination notwendig ist,
- *Notwendigkeit der Durchführung intravenöser Therapien*, wie z. B. hoch dosierte Glukokortikoide, Cyclophosphamid, Immunglobuline oder Antibiotika.
- *Immunsuppression-assoziierte Komplikationen*, z. B. opportunistische Infektionen, die eine stationäre Überwachung und Therapie erfordern,
- *Komplexe osteologische Krankheitsbilder*, wie z. B. Osteoporose mit multiplen Frakturen zur Schmerztherapie und Einleitung einer spezifischen osteologischen Therapie,
- *Geriatrische Syndrome* wie Sarkopenie, Frailty oder erhöhte Sturzgefahr, die eine umfassende funktionelle und soziale Betreuung notwendig machen,
- *Indikation zur multimodalen rheumatologischen Komplexbehandlung* einschließlich psychologischer Begleitung, Physio- und Ergotherapie sowie Schulungen.

**Tab. 2:** Mögliche Kontextfaktoren als Indikatoren einer akutstationären Betreuung/Weiterbildung

1.	Hohe Schmerzintensität, ambulant nicht beherrschbar
2.	Akut aufgetretene Fähigkeitsstörung in den Punkten Selbsthilfefähigkeit, Mobilität, Kognition und/oder Emotion beim älteren Rheumapatienten – Erstmanifestation wie auch Rezidiv einer Vaskulitis bzw. einer Kollagenose
3.	Therapiekomplikationen
4.	Drohende oder progrediente Organbeteiligung
5.	Infektionen zur intravenösen Antibiotikatherapie
6.	Krankheitsschub bei gleichzeitig vorliegender Multimorbidität
7.	Notwendigkeit einer intensivmedizinischen Versorgung
8.	Notwendigkeit der gleichzeitigen gemeinsamen Behandlung mit einer weiteren Fachdisziplin
9.	Sarkopenie
10.	Frailty
11.	Geriatrische Rheumapatienten
12.	Indikation zur Durchführung der multimodalen rheumatologischen Komplexbehandlung
13.	Therapeutische Intervention einer Komorbidität, z. B. konservative Schmerzbehandlung und Start einer spezifischen Osteoporosetherapie bei neu aufgetretenen Frakturen (auch nach stattgehabter operativer Intervention)

Diese Kontextfaktoren sind nicht als „weiche“ Kriterien zu verstehen, sondern stellen integrale Bestandteile moderner medizinischer Entscheidungsfindung dar. Sie ermöglichen eine patientenzentrierte Versorgung, die sowohl auf die medizinischen Bedürfnisse als auch auf die individuelle Lebenssituation eingeht.

Angesichts der Weiterentwicklung in der Rheumatologie und klinischen Immunologie ist eine kontinuierliche Überprüfung und Erweiterung der stationären Aufnahmekriterien erforderlich. Insbesondere neue Krankheitsentitäten und Therapieansätze – wie autoinflammatorische Erkrankungen, primäre und sekundäre Immundefekte sowie zelluläre Therapien – machen eine präzisierte Anpassung notwendig. Die Behandlung schwerer Infektionen unter immunsuppressiver Therapie, Diagnostik von seltenen Systemerkrankungen oder Therapien mit potenziell lebensbedrohlichen Nebenwirkungen sind Beispiele für Szenarien, die eine stationäre Überwachung unabdingbar machen.

Für die Behandlung entzündlich rheumatischer Systemerkrankungen (insbesondere bei bedrohlichen Organbeteiligungen) spielen interdisziplinäre Fallkonferenzen (z. B. Entzündungsboard, ILD-Board) zukünftig eine immer entscheidendere Rolle.

## Die stationäre Weiterbildung: Notwendigkeit und Struktur

Die Weiterbildung im Fachgebiet der Rheumatologie ist untrennbar mit der Versorgung komplexer systemischer Erkrankungen verbunden, deren Diagnostik und Therapie in vielen Fällen nur im stationären Rahmen möglich ist. Es ist selbstredend, dass man an den komplizierten Verläufen schwerkranker Patienten am meisten lernt: die frühzeitige Erkennung möglicher Komplikationen, die variable und hochkomplexe Ausprägung entzündlicher rheumatologischer Systemerkrankungen, die abwägende Diskussion der Fälle mit erfahrenen Fachärztinnen bei der Visite unter Einschluss aller stationär schnell verfügbaren diagnostischen Befunde, letztlich auch die Bestätigung oder Widerlegung von Arbeitshypothesen über die Tage des stationären Aufenthalts machen einen guten und erfahrenen Facharzt aus. Dies gilt für jedes Fach der Medizin, unsere Patienten haben Anspruch auf eine Betreuung durch optimal weitergebildete Ärztinnen, das muss auch der Anspruch des Weiterbildungssystems in Deutschland sein. Die Musterweiterbildungsordnung (MWBO) aus dem Jahr 2018 [10] sowie das darauf aufbauende Mustercurriculum als auch das Positionspapier zur Erteilung der Weiterbildungsbefugnis der DGRh unterstreichen die Bedeutung eines umfassenden Kompetenzerwerbs [11, 12], der durch Weiterbildung im ambulanten Sektor nicht vollumfänglich zu vermitteln ist. Die stationäre Weiterbildung ermöglicht es angehenden Fachärztinnen und Fachärzten, zentrale klinische Fähigkeiten zu erlernen, die für die Versorgung von Patienten mit potenziell lebensbedrohlichen entzündlich rheumatischen Systemerkrankungen erforderlich sind. Dazu zählen insbesondere die Einschätzung und Behandlung akuter Krankheitsschübe mit Multiorganbeteiligung, die Einleitung komplexer immunologischer Therapien, das Management von Komplikationen unter immunsuppressiver Behandlung sowie die differenzierte Diagnostik seltener Krankheitsbilder wie autoinflammatorischer Syndrome oder primärer und sekundärer Immundefekte.

Moderne Therapiekonzepte, die derzeit für refraktäre Autoimmunerkrankungen in spezialisierten stationären Zentren zur Anwendung kommen, aber zum Standardrepertoire in der Rheumatologie aufwachsen werden, erfordern fundierte Kenntnisse hinsichtlich Indikation, Durchführung, Überwachung und Nebenwirkungsmanagement. Die Vermittlung dieser Kompetenzen ist im ambulanten Versorgungsalltag nicht abbildbar und setzt zwingend die Einbindung in ein stationäres klinisches Umfeld mit entsprechender Infrastruktur und Supervision voraus.

Zudem hat die demografische Entwicklung mit zunehmendem Anteil älterer und multimorbider Patienten die Versorgungsrealität in der Rheumatologie verändert. Die Behandlung dieser Patientengruppe verlangt eine ausgeprägte Fähigkeit zur interdisziplinären Koordination und zusätzliches, geriatrisch rheumatologisches Fachwissen (z. B. zu den Themen Frailty oder Sarkopenie), die häufig nur im stationären Setting strukturiert erlernt und geübt werden. Es besteht aber die Möglichkeit, dass auch junge Patientinnen und Patienten mit akuten entzündlichen rheumatischen Erkrankungen einer stationären Versorgung bedürfen. Obwohl die Betroffenen in der Regel einen besseren Allgemeinstatus aufweisen als ältere Personen, ist dennoch eine hohe Morbidität und Mortalität zu beobachten. Darüber hinaus bedingt die psychosoziale Belastung, die mit einer neu diagnostizierten chronischen Erkrankung in jungen Jahren einhergeht, in vielen Fällen eine multidisziplinäre Betreuung, die ambulant nicht im erforderlichen Umfang realisiert werden kann. Die Behandlung komplexer Fälle erfordert zudem eine enge Kooperation mit anderen Fachdisziplinen, wie etwa Nephrologie, Pulmologie, Kardiologie, Infektiologie, Geriatrie oder Psychologie. Eine interdisziplinäre Zusammenarbeit ist für die Behandlung dieser Patienten unabdingbar und erfordert Zeit und Ressourcen. Die interdisziplinäre Diskussion der Fälle ist ein integraler Bestandteil einer optimierten stationären Weiterbildung.

Ein besonderes Qualitätsmerkmal stationärer Weiterbildung ist die Möglichkeit zur engmaschigen, kontinuierlichen Supervision [13]. Im Rahmen des stationären Alltags stehen den Weiterzubildenden erfahrene Fachärztinnen und Fachärzte zur Seite, mit denen komplexe diagnostische und therapeutische Entscheidungen gemeinsam reflektiert werden können. Darüber hinaus bietet das stationäre Umfeld vielfältige Lernanlässe wie interdisziplinäre Fallkonferenzen (z. B. Entzündungsboards, ILD-Boards, Radiologiekonferenzen), die Interpretation komplexer Befunde sowie die Planung multimodaler Therapiekonzepte, wie sie im Rahmen der rheumatologischen Komplexbehandlung zum Einsatz kommen.

Ein weiterer zentraler Aspekt der stationären Weiterbildung ist die praxisnahe Schulung in sektorübergreifender, also auch ambulanter Versorgung. Die Integration in Strukturen wie der ambulanten spezialfachärztlichen Versorgung (ASV), Medizinischen Versorgungszentren (MVZ) oder fachärztlichen Ermächtigungsambulanzen kann nur gelingen, wenn Weiterzubildende lernen, stationäre Therapieentscheidungen sinnvoll in langfristige ambulante Versorgungspläne zu überführen. Dazu gehört auch die Organisation von Überleitungen, das Schnittstellenmanagement mit Hausärztinnen und Hausärzten sowie die Mitgestaltung ambulanter Nachsorgeprozesse nach stationären Behandlungen.

Zusammenfassend lässt sich festhalten, dass die stationäre Weiterbildung eine unverzichtbare Säule in der Weiterbildung zur Fachärztin oder zum Facharzt für Rheumatologie darstellt. Nur durch eine strukturierte Einbindung in stationäre Versorgungseinheiten ist es möglich, die wachsenden Anforderungen an Diagnostik, Therapie, Interdisziplinarität und Versorgungskontinuität angemessen zu erfüllen. Eine weitere Reduktion stationärer Weiterbildungsangebote würde nicht nur die Qualität der Patientenversorgung gefährden, sondern langfristig auch die Verfügbarkeit einer rheumatologischen Fachärzteschaft.

## Versorgungspolitische Forderungen und Lösungsansätze

Zur Sicherstellung der akut-rheumatologischen Versorgung ist der Erhalt und Ausbau stationärer Weiterbildungskapazitäten essenziell. In einem entsprechenden Memorandum fordert die DGRh 3 Fachärzt:innen pro 100.000 Einwohner:innen, was einen Mehrbedarf von 700 Fachärzt:innen für Rheumatologie bedeutet [1]. Aufgrund der Altersstruktur müssen in den nächsten Jahren zusätzlich ca. 50 % der Facharztpositionen in der Rheumatologie neu besetzt werden, was einen weiteren Bedarf an zukünftigen Fachärztinnen und Fachärzten für Rheumatologie bedeutet [14]. In diesem Zusammenhang muss die Weiterbildung vom Gesetzgeber an den Versorgungsbedarf der Bevölkerung angepasst werden.

Laut einer Umfrage der Kommission Fort- und Weiterbildung der DGRh befinden sich 81,8 % der Weiterbildungsstellen im stationären Setting [15].

Der Weiterbildungsaufwand ist im DRG-System (Diagnosis Related Groups – Fallpauschalen) bislang unzureichend berücksichtigt [15]. Aktuell sind Abteilungen mit einem hohen Umsatz bzw. Gewinn mit einem deutlich besseren Personalschlüssel inklusive Weiterbildungsstellen ausgestattet [15]. Eine Entkopplung von Krankenhausfinanzierung und Weiterbildung ist daher zu fordern, wie sie in anderen europäischen Ländern bereits existiert. Der Hauptanteil der Weiterbildungsstellen befindet sich an Universitätskliniken und Kliniken eines öffentlichen bzw. konventionellen Trägers [15]. In diesem Zusammenhang ist der Ausbau weiterer universitären rheumatologischer Abteilungen als sinnvoll anzusehen.

Zusätzlich sollten sektorenübergreifende Weiterbildungskonzepte etabliert werden, die eine eigenständige Vergütung des Weiterbildungsaufwandes vorsehen [15]. In diesem Zusammenhang sollte eine Finanzierung ambulanter rheumatologischer Weiterbildungsstellen durch die kassenärztlichen Vereinigungen – analog zur Allgemeinmedizin – erfolgen [16].

Im Vergleich zu anderen europäischen Staaten wird die Finanzierung der ärztlichen Weiterbildung häufig unabhängig von der jeweiligen Versorgungsstruktur gestaltet. Häufig erfolgt die Weiterbildung bedarfsorientiert. Ein direkter Vergleich mit Deutschland ist jedoch oft schwierig, da die Strukturen des Gesundheitswesens, die Rolle der Fachärzte in der Primärversorgung und die sektorale Trennung zwischen ambulanter und stationärer Versorgung erheblich variieren. Dennoch können solche internationalen Modelle wichtige Impulse für die Weiterentwicklung der Weiterbildungs- und Versorgungsstrukturen in Deutschland liefern, insbesondere im Hinblick auf eine entkoppelte, versorgungsorientierte Finanzierung von Weiterbildungsstellen.

## Schlussfolgerung

Die aktuelle Krankenhausreform in Deutschland sowie die zunehmende Ambulantisierung der medizinischen Versorgung haben zu einem Rückgang stationärer Weiterbildungskapazitäten in vielen Fächern der Medizin, auch der Rheumatologie geführt. Gleichzeitig nimmt die medizinische Komplexität entzündlich rheumatischer Erkrankungen stetig zu. Diese manifestieren sich heute nicht mehr allein als Erkrankungen des Bewegungsapparates, sondern auch in Form von Kollagenosen, Vaskulitiden und autoinflammatorischen Erkrankungsbildern, welche als inflammatorische Systemerkrankungen mit potenziell lebensbedrohlicher Organbeteiligung zu verstehen sind. Schwere Verläufe, akute Komplikationen und innovative Therapiekonzepte wie zellbasierte Immuntherapien sowie die Versorgung vulnerabler Patientengruppen – insbesondere älterer und multimorbider Menschen – machen eine engmaschige, interdisziplinär koordinierte und häufig nur stationär durchzuführende Betreuung erforderlich.

Vor diesem Hintergrund ist die stationäre Versorgung nicht nur für die bestmögliche Behandlung dieser Patientinnen und Patienten unverzichtbar, sie stellt auch eine essenzielle Grundlage für die Facharztweiterbildung dar. Der Erwerb entscheidender klinischer Kompetenzen – von der Differenzialdiagnose seltener Systemerkrankungen über die Beherrschung schwerer Krankheitsverläufe bis hin zur Implementierung moderner Therapiestrategien – kann nicht adäquat in rein ambulanten Strukturen erfolgen. Die stationäre Weiterbildung ermöglicht zudem eine strukturierte Supervision und den Einblick in sektorenübergreifende Versorgungsprozesse, wie sie im Rahmen von ASV-Strukturen und interdisziplinären Fallkonferenzen erforderlich sind.

Um eine qualitativ hochwertige rheumatologische Versorgung auch zukünftig sicherzustellen, muss die stationäre Weiterbildung strukturell erhalten, fachlich weiterentwickelt und politisch gefördert werden. Andernfalls drohen eine Verstärkung des vorhandenen Versorgungsdefizits bei einer gleichzeitig wachsenden Zahl komplex erkrankter Patientinnen und Patienten sowie eine Schwächung des ärztlichen Nachwuchses in einem zentralen internistischen Fachgebiet. Stationäre rheumatologische Strukturen sind daher nicht nur medizinisch und in Bezug auf die ärztliche Weiterbildung erforderlich – sie sind eine Voraussetzung für die Zukunftsfähigkeit der rheumatologischen Versorgung in Deutschland.

## Fazit für die Praxis

- Entzündlich rheumatische Erkrankungen mit Multiorganbeteiligung und hoher klinischer Komplexität erfordern weiterhin stationäre Diagnostik, Therapie und Überwachung – insbesondere bei akuten Verläufen, Komplikationen, Komorbiditäten oder unter neuen immunmodulierenden Therapien.
- Der Erwerb wesentlicher klinischer Kompetenzen, insbesondere im Umgang mit schweren, seltenen oder lebensbedrohlichen Verläufen, setzt eine strukturierte stationäre Weiterbildung voraus.
- Die Zukunftsfähigkeit der rheumatologischen Versorgung hängt wesentlich davon ab, dass stationäre Weiterbildungs- und Versorgungsstrukturen erhalten bleiben.

## Abkürzungen

CAR: Chimärer Antigenrezeptor
DGRh: Deutsche Gesellschaft für Rheumatologie und Klinische Immunologie
KHVVG: Krankenhausversorgungsverbesserungsgesetz
KOBRA: Kontinuierliches Outcome Benchmarking in der Rheumatologischen Akutversorgung
TCE: T‑Cell-Engager
VRA: Verband Rheumatologischer Akutkliniken
